# The role of TMS 12 in the staphylococcal multidrug efflux protein QacA

**DOI:** 10.1093/jac/dkad121

**Published:** 2023-04-26

**Authors:** Abolfazl Dashtbani-Roozbehani, Mohsen Chitsaz, Melissa H Brown

**Affiliations:** College of Science and Engineering, Flinders University, Bedford Park, SA 5042, Australia; College of Science and Engineering, Flinders University, Bedford Park, SA 5042, Australia; College of Science and Engineering, Flinders University, Bedford Park, SA 5042, Australia

## Abstract

**Objectives:**

To elucidate the importance of a region in QacA predicted to be important in antimicrobial substrate recognition.

**Methods:**

A total of 38 amino acid residues within or flanking putative transmembrane helix segment (TMS) 12 of QacA were individually replaced with cysteine using site-directed mutagenesis. The impact of these mutations on protein expression, drug resistance, transport activity and interaction with sulphhydryl-binding compounds was determined.

**Results:**

Accessibility analysis of cysteine-substituted mutants identified the extents of TMS 12, which allowed for refinement of the QacA topology model. Mutation of Gly-361, Gly-379 and Ser-387 in QacA resulted in reduced resistance to at least one bivalent substrate. Interaction with sulphhydryl-binding compounds in efflux and binding assays demonstrated the role of Gly-361 and Ser-387 in the binding and transport pathway of specific substrates. The highly conserved residue Gly-379 was found to be important for the transport of bivalent substrates, commensurate with the role of glycine residues in helical flexibility and interhelical interactions.

**Conclusions:**

TMS 12 and its external flanking loop is required for the structural and functional integrity of QacA and contains amino acids directly involved in the interaction with substrates.

## Introduction

MDR efflux pumps are recognized as a major mechanism of antimicrobial resistance in pathogenic bacteria and one of the principal causes of infection treatment failure.^1,2^ By extruding a broad range of structurally different toxic compounds and hence decreasing their intracellular concentration, efflux pumps enable bacteria to survive.^3,4^ The widespread use of biocides such as quaternary ammonium compounds (QACs) (e.g. benzalkonium and chlorhexidine) for disinfection and antiseptic practice in healthcare and community settings has required bacteria within these environments to employ various tactics to limit their exposure to such antimicrobials.^5–8^ The nosocomial pathogen *Staphylococcus aureus* achieves this through the action of the QacA efflux pump.^9,10^ Prevalence of *qacA* among *S. aureus* clinical isolates fluctuates depending on their geographical location, varying from 10% to 80%.^11^ Highlighting the importance of this resistance determinant, an increasing trend in *qacA* prevalence in clinical staphylococcal isolates has been observed in the USA and Asian countries,^7^ facilitated by it being a plasmid-borne efflux pump.^8^

The 514 amino acid QacA protein belongs to the major facilitator superfamily (MFS) of transport proteins, the largest grouping of secondary active transporters that are present in all classes of living organisms. Three families within the MFS exclusively contain drug-efflux systems, designated DHA1–3 for drug:H^+^ antiport, whose protein members contain 12 (DHA1 and 3) or 14 (DHA2) α-helical transmembrane segments (TMSs). QacA is a member of the DHA2 family and mediates resistance to a wide range of cationic antimicrobials commonly used as antiseptics and disinfectants.^12–16^ Through structure–function analyses primarily by cysteine-scanning mutagenesis and chemical labelling techniques, a large number of QacA residues critical for the transport of multiple substrates has been identified.^12,15,17–21^ Mutagenic and competition studies have led to the hypothesis that monovalent substrates either share a common binding site or have unique but overlapping binding sites, whereas bivalent cations interact at a distinct site(s).^14,22^ This was supported by binding studies with purified QacA that identified two distinct sites that TMS 1 and TMS 13 contributed to.^15^ Furthermore, TMS 10 forms an integral part of the bivalent substrate-binding pocket of QacA, with Asp-323 playing a critical role in QacA resistance activity against bivalent cations.^18^ Notably, QacB, a paralogue of QacA, does not confer resistance to bivalent cations, presumably due to the presence of a neutral residue at position 323.^12,14^ However, acidic residue substitutions at position 377 (in TMS 12) restored a significant overall degree of bivalent drug resistance in QacA after the neutralization of Asp-323 leading to the speculation that TMS 12 is involved in interactions with bivalent substrates.^19^

To ascertain if residues within TMS 12 are important for the MDR ability of QacA, 38 amino acid residues from 361 to 402, which includes putative TMS 12 and its adjacent loop regions, were mutated and the functional impact of their substitution analysed. Resistance profiling, fluorescence-based transport assays, and interaction with sulphhydryl-binding compounds identified residues within the target region that play a functional role in substrate binding and the translocation pathway by QacA. Moreover, the exact boundaries of TMS 12 and the microenvironment of each residue within the QacA protein were determined.

## Materials and methods

### Bacterial strains, plasmids and growth conditions

The *Escherichia coli* strain DH5α^23^ was used as the bacterial host for mutant construction, heterologous protein expression and functional assays. A previously constructed pBlueScript II SK-based *qacA* clone with a 6 × His-tag encoding sequence at its 3′ end (pSK7201)^18^ was the template for construction of QacA site-directed mutants. Cultures of *E. coli* cells were grown at 37°C in LB medium supplemented with 100 mg/L ampicillin where appropriate.

### Site-directed mutagenesis

Each target residue in QacA was individually substituted with cysteine using site-directed mutagenesis based on the QuikChange^™^ method (Stratagene).^20^ Details of the primer pairs (Sigma–Aldrich) containing a cysteine codon along with a silent mutation for restriction site screening are provided in Table S1 (available as Supplementary data at *JAC* Online). DNA sequencing was conducted (Australian Genomic Research Facility, Adelaide) to verify the presence of the incorporated cysteine as well as the absence of spurious mutations within the entire *qacA* sequence.

### Preparation of membrane vesicles and determination of QacA mutant expression levels

Membrane vesicles were isolated as previously described.^18,21^ Briefly, cells carrying pBlueScript-based QacA variants were lysed by one passage through a cell disrupter (Constant Systems) at 30 000 psi. Membrane vesicles were isolated by centrifugation (134 000 × g, 1 h, 4°C) and resuspended in buffer [20 mM Tris-HCl (pH 7.5), 300 mM NaCl, 10% (v/v) glycerol]. Protein concentrations were quantified using a bicinchoninic acid (BCA) assay (Bio-Rad). Equal amounts of protein were loaded onto a 10% SDS-PAGE gel and western blot analysis was performed using a primary rabbit anti-6 × His-tag antibody (Rockland Immunochemicals, 1:5000 dilution) and a secondary goat-anti-rabbit IgG conjugated to horseradish peroxidase (Bio-Rad, 1:10 000 dilution).^17,20^ Blots were scanned using a ChemiDoc^™^ MP Imaging System (Bio-Rad) and analysed by Image Lab software (Bio-Rad).

### Fluorescein maleimide (FM) labelling assay

FM labelling of QacA mutant proteins containing a single cysteine (QacA does not contain any native cysteine residues) was determined essentially as outlined elsewhere.^18,21^ In short, FM was added to membrane vesicles (250 μg) to a final concentration of 0.25 mM and incubated at 37°C for 10 min. Proteins were solubilized with 10% *n*-dodecyl β-d-maltoside (DDM) and the QacA protein bound to ProBond^™^ Nickel-Chelating Resin (Life Technologies) and eluted using a buffer [20 mM Tris-HCl (pH 7.5), 10% (v/v) glycerol, 400 mM imidazole, 0.1% (w/v) DDM]. Purified protein samples (2 μg) were resolved on 10% SDS-PAGE and scanned using a Gel-Doc system (Bio-Rad) to detect the associated fluorescein labels. The gels were also stained with Coomassie blue to visualize total QacA protein. All images were analysed using Image Lab software (Bio-Rad) to obtain the relative to WT fluorescence intensity for each mutant protein. To determine the influence of substrate binding on the FM labelling reaction, membrane vesicles were preincubated in the absence or presence of a QacA substrate (2 mM) at room temperature for 5 min prior to the addition of FM.

### MIC analysis

Susceptibilities of all QacA mutants were determined using the standard agar dilution method described previously.^21^ Antimicrobial compounds were chosen as representatives of different chemical classes and were added to Mueller–Hinton agar plates in the following concentration ranges: ethidium (25 to 450 mg/L; increments of 25 mg/L), rhodamine 6G (200 to 1200 mg/L; increments of 50 mg/L), benzalkonium (20 to 150 mg/L; increments of 10 mg/L), dequalinium (50 to 450 mg/L; increments of 25 mg/L), chlorhexidine (0.5 to 12 mg/L; increments of 0.5 mg/L) or pentamidine (60 to 400 mg/L; increments of 20 mg/L). Freshly transformed bacteria were grown at 37°C for 24–48 h and the MIC determined as the lowest antimicrobial concentration that fully inhibited visible growth. Additional validation of the drug resistance phenotype was performed using a plate dilution assay by spotting 4 μL of a series of 10-fold dilutions of bacteria harbouring WT QacA and QacA mutants on Mueller–Hinton plates with or without the addition of substrates.

### Fluorescent transport assays

The transport of ethidium (monovalent substrate) and DAPI (bivalent substrate) was measured fluorimetrically in *E. coli* DH5α cells as previously described.^14,18^ Briefly, freshly transformed cells carrying the WT QacA or its mutants were grown to OD_600 _= 0.6 and resuspended in 20 mM HEPES buffer (pH 7.0). Cells were loaded with 15 μM ethidium or 10 μM DAPI in the presence of 10 μM carbonyl cyanide *m*-chlorophenyl hydrazone, a protonophore. Energy-starved and substrate-loaded cells were energized by adding 125 mM sodium formate and fluorescence was measured for at least 5 min at excitation and emission wavelengths of 530 and 590 nm, for ethidium, and 364 and 454 nm, for DAPI, with a fluorescence spectrometer (LS 55, Perkin-Elmer). To evaluate the effect of maleimide binding on the efflux of DAPI, cells carrying cysteine-substituted QacA mutants were pretreated with or without 5 mM *N*-ethylmaleimide (NEM), a sulphhydryl-binding compound, for 20 min at 37°C prior to substrate loading.

## Results and discussion

### Construction of QacA cysteine mutants and their protein expression levels

To examine the role of TMS 12 in the structure and resistance function of QacA, 38 amino acid residues spanning the predictive TMS 12 and its flanking loop regions in QacA were targeted for analysis. Using site-directed mutagenesis, QacA derivatives were constructed where residues were individually mutated to cysteine. By western blotting with an anti-6 × His antibody, a band around 50 kDa corresponding to QacA was detected from membrane preparation of cells expressing the WT and mutant QacA proteins (Figure S1). This analysis confirmed that the cysteine substitution did not have an adverse impact on QacA mutant protein expression, or insertion into the membrane, consistent with previous findings.^18^ Moreover, any functional changes observed in subsequent functional analyses of these QacA mutants could therefore be attributable to the impact of the residue substitution.

### Determination of the extents of QacA TMS 12

**Figure 1.**
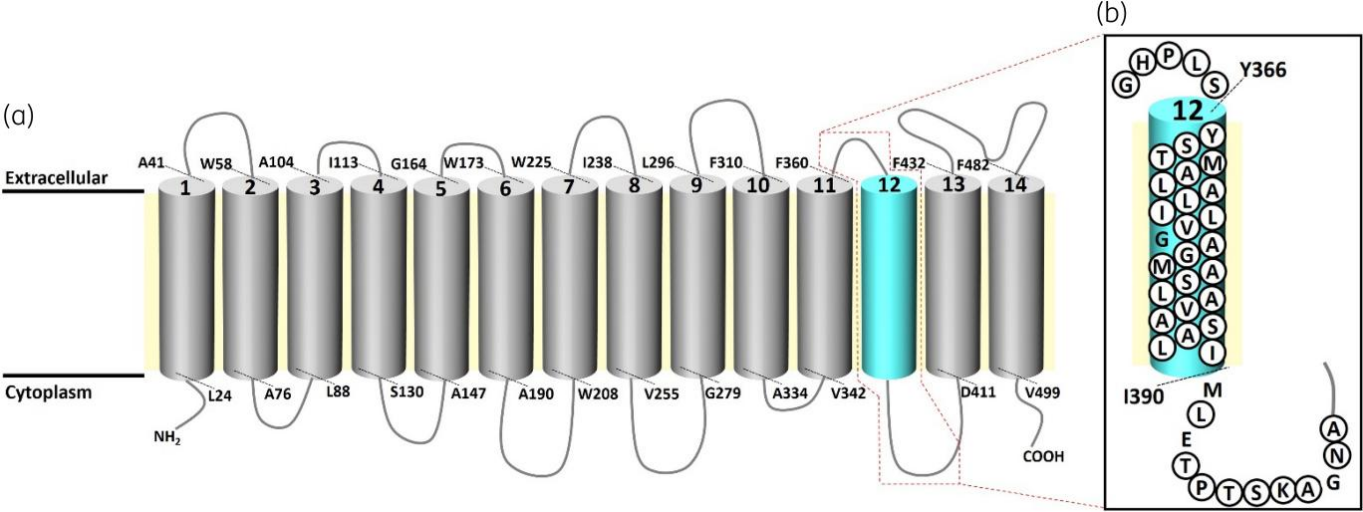
2D membrane topology model of QacA. (a) Simplified topology model of QacA with its 14 TMSs presented as cylinders labelled 1 to 14. One-letter residue names with their position numbers indicate the top and bottom extremities of TMSs previously predicted by hydropathy and solvent accessibility analyses.^12,22,24^ (b) Refined extents of TMS 12 determined, based on analysis of solvent accessibility of cysteine mutants in this study (see text for details). Residues in circles were mutated in this study and others (Gly-377, Met-391, Glu-393 and Gly-400) were analysed in previous studies.^19,21^ This figure appears in colour in the online version of *JAC* and in black and white in the print version of *JAC*.

The placement of residues within the TMS of QacA, including TMS 12, have been previously predicted (Figure 1a) based on hydropathy profile analysis and gene fusion studies.^12,22,24^ However, the termini of TMS 12 remained to be experimentally validated. Labelling of cysteine mutants with sulphhydryl-reactive reagents such as FM has been used to experimentally probe whether a residue is located in the hydrophobic core of membrane bilayer (unreactive to labelling) or in a surface hydrophilic environment (able to be labelled) and therefore enabling the assignment of TMS in transporters,^25–28^ including QacA.^15,18^

The FM reactivity levels of the 38 cysteine-substituted QacA mutants in this study were compared with that of a QacA P309C mutant, previously shown to be highly reactive with FM and therefore located outside of the membrane environment.^18^ The QacA Tyr-366, Ser-367, Thr-368 and Met-369 mutants showed only weak reactivity with FM, indicating the location of these residues is more likely to be inside the membrane bilayer. In contrast, high reactivity with FM was detected with QacA Gly-361, His-362, Pro-363, Leu-364 and Ser-365 mutants, confirming their placement in the loop connecting TMS 11 and TMS 12 (Figure S2). Furthermore, the solvent-accessibility analysis determined that Tyr-366 and Ile-390 are located at the extracellular and cytosolic ends of TMS 12, respectively. Therefore, these experimental data enabled the refinement of the TMS 12 extents in the QacA model (Figure 1b), showing that TMS 12 is a relatively long helix composed of 25 amino acid residues. Moreover, the solvent-accessibility data revealed that Ala-384, Ser-387 and Ala-388, although embedded in the TMS 12, somewhat strongly reacted with FM, in contrast to other residues buried in the hydrophobic core of TMS 12 (Figure S2). This suggests that Ala-384, Ser-387 and Ala-388 might be part of the accessible substrate translocation pathway.

### Resistance capacities of QacA mutants

Resistance profiling by MIC analyses was used to assess the functional importance of residues in the target region. QacA-mediated resistance to a panel of six antimicrobial compounds, chosen as representative substrates from different chemical classes, was determined. These include the monovalent dyes (ethidium and rhodamine 6G) and QAC (benzalkonium), and bivalent QAC (dequalinium), biguanidine (chlorhexidine) and diamidine (pentamidine). The resistance levels conferred by each QacA mutant to these substrates was compared with WT QacA and are presented as percentages in Table 1. As can be seen, the majority of the mutants showed similar or only slightly reduced/increased levels of resistance to each of the antimicrobials tested in comparison with the WT QacA (indicative of 100% resistance activity), implying these are not important in the resistance mechanism. Substitutions of TMS12-embedded Gly-379 and Ser-387 resulted in ≥50% reduced resistance to the three bivalent compounds whereas the substitution of Gly-361 (situated in extracellular loop 11–12) only impacted on resistance to the bivalent substrate pentamidine (Table 1, bold type). Additionally, agar plate dilution assays for cells harbouring WT QacA and QacA mutants confirmed that these mutations caused substantial reduction of resistance to the aforementioned antimicrobial compounds (Figure S3). Together these data clearly suggest that Gly-361, Gly-379 and Ser-387 are functionally important in QacA, participating in an aspect of substrate binding, translocation and/or conformational transitions during flux of selective substrates. Moreover, QacA S387C was the only mutant that showed a substantial reduction of resistance to the monovalent compound benzalkonium. Thus, Ser-387 must be positioned such that it plays a role in resistance to both monovalent and bivalent substrates. This may be achieved through a general reduction in pump function rather than through a specific interaction with individual substrates.

**Table 1.** Resistance profiles of QacA mutants

Mutation	Location of mutation^a^	Relative MICs of QacA mutants (% WT QacA)^b^					
		Monovalent substrates			Bivalent substrates		
		Et	R6G	BK	DQ	CH	PE
WT QacA^c^		100 (250)	100 (800)	100 (100)	100 (300)	100 (7)	100 (240)
No QacA		20	31	30	25	14	33
**G361C**	Loop 11–12	100	100	100	75	79	**50**
H362C	Loop 11–12	100	112	100	83	100	83
P363C	Loop 11–12	100	100	90	83	71	83
L364C	Loop 11–12	100	100	90	83	71	100
S365C	Loop 11–12	100	113	100	83	100	83
Y366C	Loop 11–12	100	113	100	125	100	83
S367C	Loop 11–12	100	113	100	125	100	83
T368C	Loop 11–12	100	113	90	100	100	83
M369C	TMS 12	100	100	80	100	100	92
A370C	TMS 12	100	100	100	125	100	83
L371C	TMS 12	100	100	100	100	100	100
A372C	TMS 12	100	100	100	100	86	100
L373C	TMS 12	80	100	100	117	79	100
I374C	TMS 12	70	100	100	100	100	100
L375C	TMS 12	100	100	100	83	100	92
V376C	TMS 12	100	100	100	100	93	100
G377C^d^	TMS 12	40	70	100	75	50	50
A378C	TMS 12	80	100	100	100	100	100
**G379C**	TMS 12	80	100	100	**50**	**43**	**42**
M380C	TMS 12	100	100	100	100	71	83
A381C	TMS 12	80	100	100	100	100	100
S382C	TMS 12	100	100	90	100	100	100
L383C	TMS 12	100	100	100	100	100	125
A384C	TMS 12	100	119	100	100	114	125
V385C	TMS 12	100	100	100	133	100	117
A386C	TMS 12	100	119	100	125	100	117
**S387C**	TMS 12	70	81	**50**	**42**	**43**	**50**
A388C	TMS 12	100	100	100	100	100	117
L389C	TMS 12	100	100	90	83	100	100
I390C	TMS 12	100	100	100	83	107	100
M391C^d^	Loop 12–13	80	125	100	70	75	80
L392C	Loop 12–13	100	106	90	100	129	100
E393C	Loop 12–13	100	100	100	100	100	100
T394C	Loop 12–13	80	106	100	117	100	100
P395C	Loop 12–13	90	113	100	125	114	100
T396C	Loop 12–13	110	138	110	117	129	100
S397C	Loop 12–13	100	131	110	117	129	100
K398C	Loop 12–13	130	144	110	125	129	100
A399C	Loop 12–13	70	106	100	100	100	75
G400C^e^	Loop 12–13	70	80	100	90	75	60
N401C	Loop 12–13	100	94	100	108	100	133
A402C	Loop 12–13	140	131	110	125	129	133

Et, ethidium; R6G, rhodamine 6G; BK, benzalkonium; DQ, dequalinium; CH, chlorhexidine; PE, pentamidine.

Location of amino acid changes in QacA is based on the refined QacA topology model (Figure 1).

Numbers indicate relative resistance level of the mutants in comparison with the WT QacA (indicative of 100% resistance activity). MIC values determined by a standard agar dilution method, where *E. coli* DH5α cells expressing WT and mutant QacA proteins were grown on LB agar supplemented with different concentrations of individual compounds. Bold font indicates ≤50% MIC compared with WT QacA. All MIC data are representative of at least three independent experiments and are presented as percentage value relative to WT QacA.

MIC value (mg/L) for WT QacA against each substrate is shown in parentheses.

Previously constructed mutants included here for comparison.^19,21^

Another noteworthy observation is that among the mutants tested, three mutants (I374C, S387C and A399C) exhibited reduced MICs of ethidium (Table 1) suggesting an involvement in interactions with this substrate. Therefore, together with above-mentioned results, it can be concluded that TMS 12 is involved in the interaction with both monovalent and bivalent cationic substrates, similar to what has been found previously with TMS 10.^18^ Presumably, TMS 12 forms part of the binding site that overlaps between mono- and bivalent cationic substrates.

### Spatial orientation of the identified important residues

**Figure 2.**
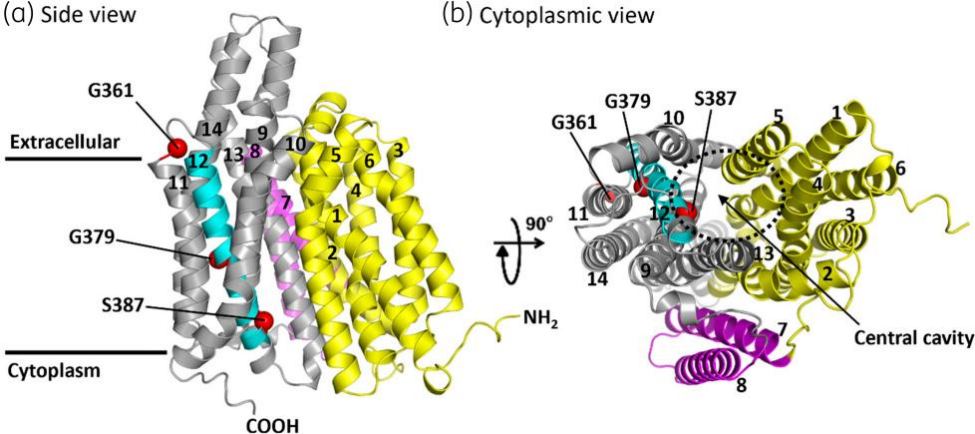
3D predicted structure of QacA from *S. aureus*. AlphaFold-predicted model of QacA in the inward-open state, showing the spatial orientation of the 14 TMSs of QacA from the side and cytoplasmic view. (a) The model displays 2-fold symmetrical domains with TMSs 1–6 in the N-terminal domain (yellow) and TMSs 9–14 in the C-terminal domain (grey), connected by TMS 7 and 8 (magenta). TMS 12 (Tyr-366 to Ile-390), the subject of this study, is highlighted in cyan, and residues identified as functionally important are shown as red spheres. (b) Bottom view from the cytoplasmic side of QacA showing the central cavity. TMSs 1, 2, 4, 5, 9, 10, 12 and 13 are directly lining the cavity while TMSs 3, 6, 11 and 14 are farther away. TMSs 7 and 8, which connect the N- and C-domains, are situated at the side of the 12-TMS structural fold. Among the identified functional residues, only Ser-387 is oriented towards the central cavity. The figures were created with PyMol.^31^ This figure appears in colour in the online version of *JAC* and in black and white in the print version of *JAC*.

Recently, AlphaFold, an artificial intelligence-based method, has made a breakthrough in predicting protein structures with very high accuracy.^29,30^ The predicted model for QacA from *S. aureus* in the inward-open state was obtained from the AlphaFold Protein Structure Database (https://alphafold.ebi.ac.uk) to identify the spatial arrangements of TMS (Figure 2). The 3D QacA model exhibits a structural core that is organized into two bundles of six-TMS (N- and C-terminal domains) with the presence of a large cavity in the centre of the transporter, similar to what has been described for other MFS proteins.^32–34^ The two additional helices, particular to DHA2 MFS proteins (TMS 7 and TMS 8), drawn in the centre of the 2D model (Figure 1), are located on one side of the core. TMS 12, together with TMS 1, 2, 4, 5, 9, 10 and 13, surrounds the QacA central cavity, which is purported to be the main substrate translocation pathway, similar to the central substrate binding pocket of the MFS exporters MdfA,^35^ NorC^36^ and NorA.^37^ Positioning the identified functional residues onto the 3D model of QacA reveals their location in relation to the central cavity (Figure 2). Conspicuously, Ser-387, which is found to be both a functionally important and a solvent-accessible residue at the cytoplasmic end of TMS 12, is oriented towards the central cavity of QacA.

### Ethidium transport capabilities of QacA mutants

**Figure 3.**
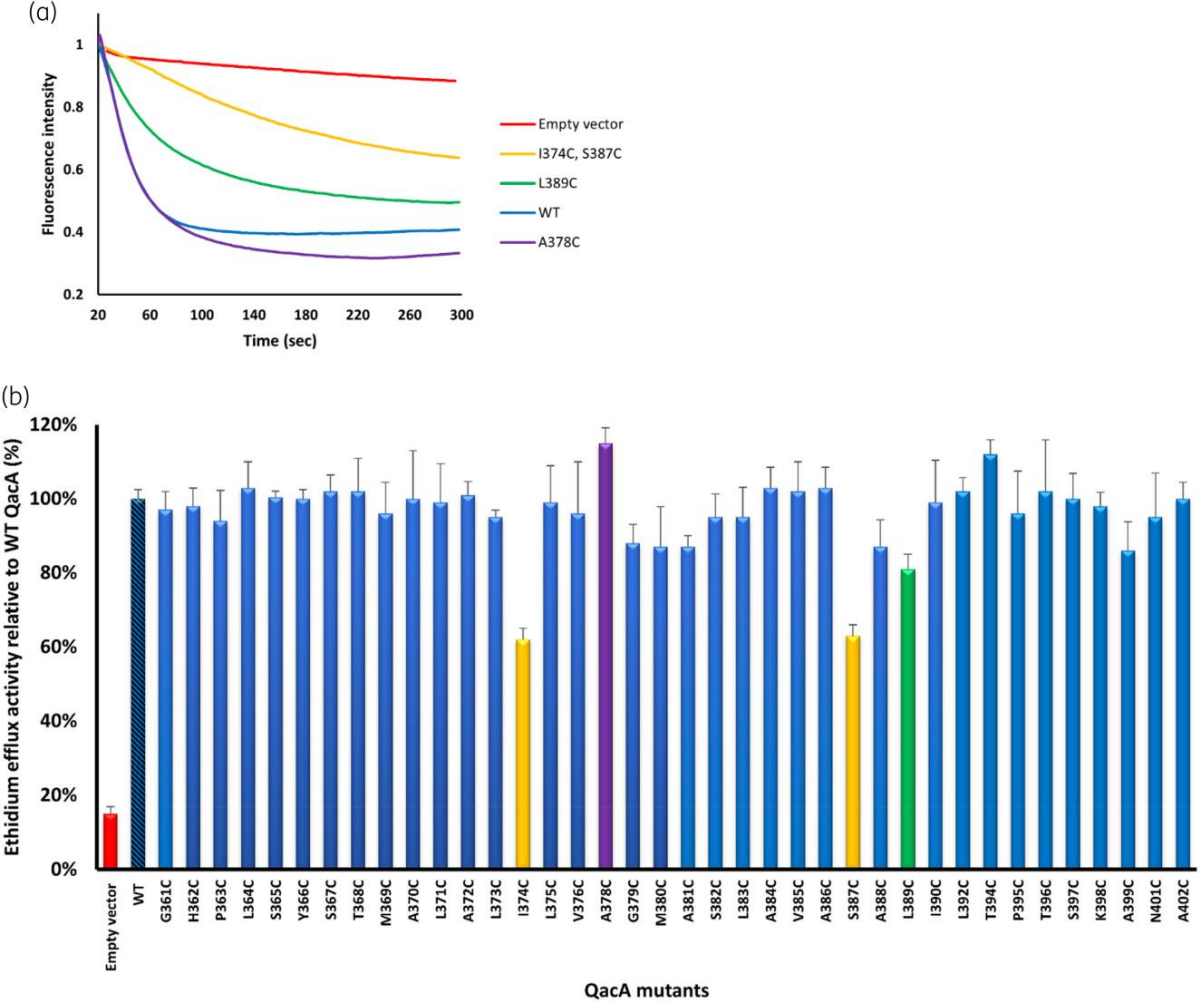
Ethidium transport activity of mutant QacA proteins. (a) Representative ethidium efflux curves of WT and four mutants including I347C and S387C with lowest, L389C with moderate, and A378C with highest efflux activities among the mutants examined in this study are shown. Other mutants had efflux curves that lie in the area between the green and purple curves. (b) Relative efflux values were calculated compared with WT QacA (the leftmost blue hatched bar), indicative of 100% efflux activity. Efflux values were taken at the end point of the efflux curves (300 s timepoint). Cells carrying the empty vector (pBlueScript II SK) are included as a negative control (red bar). Yellow, purple and green bars correspond to the representative efflux curves of QacA mutants shown in (a). Error bars represent SD of three biological replicates. This figure appears in colour in the online version of *JAC* and in black and white in the print version of *JAC*.

To augment the resistance profile analysis, the effects of residue substitutions on QacA-mediated substrate transport were examined using the monovalent fluorescent substate ethidium in an *E. coli* whole*-*cell assay. Upon accumulation inside the cells, ethidium causes an increase in fluorescence intensity, which decreases upon energization of the QacA protein (Figure 3a). The I374C and S387C QacA mutants showed impaired efflux activity (approximately 40% reduction of ethidium efflux activity compared with the parent cells). This implies that Ile-374 and Ser-387 play a role in the QacA substrate translocation process. Efflux assays of cells expressing the remaining 36 QacA mutations showed no difference to the WT QacA (Figure 3b), indicating they are not essential for the ethidium transport function of QacA. This supports the notion that the results of the ethidium transport assay of QacA mutants generally dovetails with those of the ethidium resistance assay and vice versa.

### Efflux of DAPI and effect of NEM treatment on DAPI transport in G361C, G379C and S387C QacA mutants

**Figure 4.**
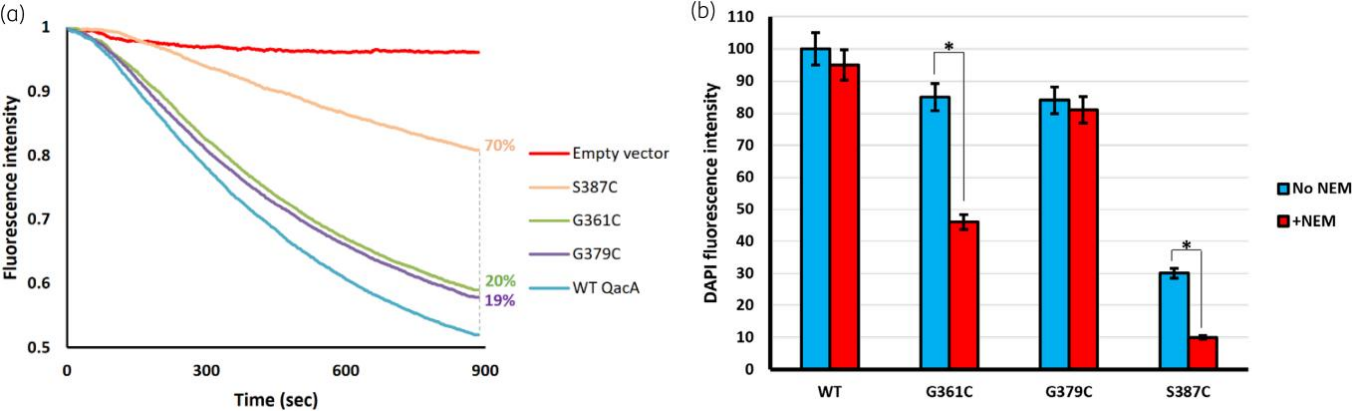
DAPI transport activity and effect of NEM treatment. (a) *E. coli* DH5α cells carrying WT QacA and G361C, G379C and S387C QacA mutants were loaded with 10 µM DAPI and efflux was measured fluorimetrically. Transport assays were performed in at least triplicate and representative results are shown. The values next to the vertical dashed line denote percentage reduction in the DAPI transport activity of each mutant compared with QacA WT level. (b) Relative efflux values of NEM-treated cells (red bars) versus non-NEM-treated cells (blue bars) compared with WT QacA without NEM (100% efflux activity) are shown. Error bars represent SD of three biological replicates. Statistical significance was determined by Student’s *t*-test. Significance (*P* < 0.05) is indicated by an asterisk. This figure appears in colour in the online version of *JAC* and in black and white in the print version of *JAC*.

Three QacA mutants, G361C, G379C and S387C, conferred ≤50% of WT levels of resistance to at least one of the tested bivalent substrates (Table 1). To determine whether these substitutions also influenced export, efflux assays with the bivalent substrate DAPI^18^ were conducted. Cells expressing G361C and G379C QacA mutants exhibited a 20% reduction in the DAPI transport activity compared with the QacA WT level (Figure 4a). This suggests that Gly-361 and Gly-379 have only a modest effect on the transport of bivalent cations. Interestingly, the S387C mutation resulted in a significant reduction (70%) in DAPI transport activity (Figure 4a) indicating that this residue plays an essential role in the transport of bivalent substrates of QacA.

Measuring transport activities after treatment of cysteine-substituted mutants with NEM (a non-fluorescent maleimide compound) has been used to gain experimental insights into the substrate-binding site and/or translocation pathway of membrane transport proteins.^38,39^ To assess whether thiol modification had an effect on the transport function of the QacA cysteine-substituted mutants at positions 361, 379 and 387, DAPI efflux studies were undertaken after treatment of the bacterial cells with NEM. As expected, NEM treatment did not impact on DAPI transport from cells carrying WT QacA, which inherently contains no cysteine residues (Figure 4b). Additionally, NEM did not influence transport from cells expressing the QacA G379C mutant. However, treatment with NEM was seen to significantly reduce DAPI efflux in cells expressing the G361C and S387C QacA mutant proteins (Figure 4b), indicating that these amino acid positions may interact directly with DAPI or the addition of the maleimide has altered the shape or architecture of the binding pocket. Indeed, binding of NEM at these positions presumably disrupts DAPI binding and/or the transport pathway due to steric hindrance between the substrate and the bulky maleimide group of NEM, akin to what has been suggested in other membrane transport proteins using a similar probing approach.^40,41^

### Influence of substrate binding on solvent accessibility

**Figure 5.**
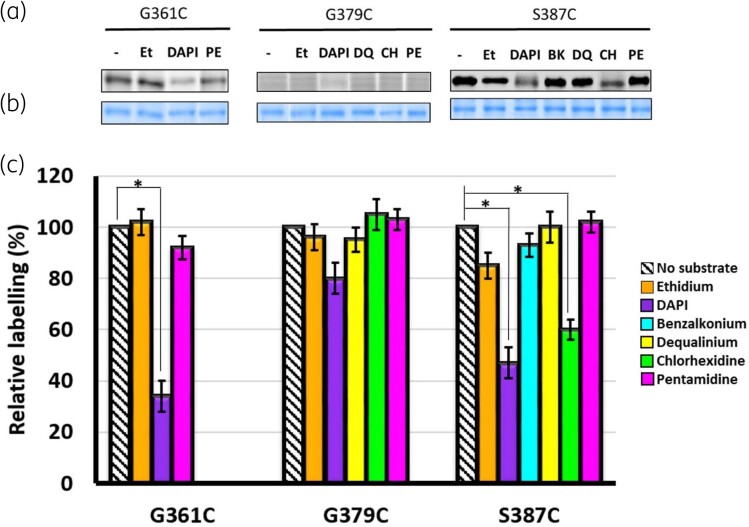
Labelling reactivity of G361C, G379C and S387C QacA mutants with FM in the presence of selected substrates. (a) Fluorescence of bands is directly related to reactivity of introduced cysteine with FM after substrate preincubation. Et, ethidium; BK, benzalkonium; DQ, dequalinium; CH, chlorhexidine; PE, pentamidine; (−), in the absence of substrate. (b) Corresponding Coomassie brilliant blue-stained bands to demonstrate equal loading of QacA on SDS-polyacrylamide gels. (c) The percent labelling intensity of the substrate-treated protein relative to the untreated protein for each mutant is shown by the height of the coloured bars. The experiment was repeated three times. Error bars represent SD of three biological replicates. Statistical significance was determined by Student’s *t*-test. Significance (*P* < 0.05) is indicated by an asterisk. This figure appears in colour in the online version of *JAC* and in black and white in the print version of *JAC*.

Cysteine labelling using maleimide derivatives after preincubation with substrates has been employed as a biochemical methodology to probe the substrate-binding site and provide conformational insights for membrane transport proteins.^42,43^ Basically, an introduced cysteine residue that is juxtaposed to the substrate-binding site(s) would be allosterically protected from maleimide modification by the addition of substrates, or substrate binding could lead to a conformational change in the protein, thereby making the cysteine residues more or less accessible to maleimide labelling.^18,42^ Against this backdrop, the effects of preincubation with selected QacA substrates on the labelling profile of the QacA G361C, G379C and S387C mutants were examined. The selection of substrates was based on the MIC results where these mutants showed lower levels of resistance against the chosen substrates (Table 1). Ethidium was an exception, since no significant reduction was observed for the MIC of ethidium; therefore, it was included as a control for all mutants. As expected, preincubation with ethidium had no effect on FM labelling of the QacA cysteine mutants. However preincubation with DAPI (Figure 5, purple) produced a significant decrease in FM labelling of G361C and S387C, suggesting that these amino acid positions are likely to be either directly involved in or located in close vicinity to the DAPI substrate-binding site. Furthermore, 40% of the QacA S387C mutant protein was protected from maleimide modification in the presence of chlorhexidine (Figure 5, green), possibly due to the direct involvement of Ser-387 in binding to chlorhexidine or that the location of this residue is in close vicinity to the chlorhexidine binding site. The addition of benzalkonium, dequalinium and pentamidine did not affect labelling profiles of the three QacA cysteine variants, suggesting that residues at positions 361, 379 and 387 are not part of the binding pockets for these substrates.

The results of DAPI binding on FM modification (Figure 5, purple) together with those from the effects of NEM treatment on DAPI transport (Figure 4b) strongly suggest that G361 and S387 are directly involved in the substrate binding site and/or translocation pathway used by DAPI. In the case of G379C, the relevant effects were marginal, implying that this residue is not directly engaged in the substrate-binding and translocation pathway. These experimental results are in agreement with the position of Gly-379 in the QacA model, where this residue is oriented away from the central cavity (Figure 2). Interestingly, multiple sequence alignment between QacA and a number of homologous multi- and single-drug transporters from the DHA1 and DHA2 members of the MFS family showed that the intramembranous glycine at position 379 is the only residue that is highly conserved (Figure S4). This suggests it is a key residue that plays a common important role in DHA family transporters. Indeed, many intramembrane conserved glycine residues have been ascribed to participate in helix–helix packing and interhelical interactions in the structure of transporters irrespective of the transport protein family.^44,45^ Taken together, it is likely that G379 in TMS 12 of QacA is structurally important for helical flexibility and interhelical interactions of TMS 12 required for transport of bivalent substrates, similar to the role previously reported for G313 in TMS 10.^18^ These TMSs are expected to have high mobility during substrate translocation, facilitated by glycine residues,^46^ as shown by spin-labelling studies for corresponding TMSs in the EmrD (12-TMS MFS) transporter.^47^ Thus, it would be reasonable to suggest that in QacA, substitution of the glycine residue at position 379 disfavours the flexibility of helix 12 and leads to a decrease in bivalent substrate flux as inferred from a substantial reduction in their MICs (Table 1). Interestingly, monovalent substrate export seems to be less dependent on helix 12 flexibility.

### Conclusions

Given the high contribution of the QacA efflux pump to antiseptics and disinfectants resistance,^8,48^ it is imperative to broaden our understanding of the structure function features of QacA, including the role of key residues in QacA drug binding and translocation. Collectively, the results in this study demonstrate that the cytoplasmic end of TMS 12 plays a direct role in the substrate-binding site and translocation pathway of QacA, in agreement with the importance of its equivalent TMS (TMS 10) in a recent structure of NorA.^37^ Additionally, the QacA AlphaFold model further consolidates the idea that the cytoplasmic end of TMS 12 is positioned at the mouth of the QacA central cavity where cytoplasmic entry of substrates, potentially, occurs. Therefore, this region could be a target for future development of inhibitors of QacA that prevent growth in combination with antimicrobial substrates.

## Supplementary Material

dkad121_Supplementary_Data

## Supplementary data

Table S1 and Figures S1, S2, S3 and S4 are available as Supplementary data at *JAC* Online.
