# Supplementary material for: The role of TMS 12 in the staphylococcal multidrug efflux protein QacA
Source: J Antimicrob Chemother. 2023 Apr 26;78(6):1522–31. doi: 10.1093/jac/dkad121 (PMC10269129; doi:10.1093/jac/dkad121)
Supplement: dkad121_Supplementary_Data [file dkad121_supplementary_data.docx]

## Figure S1


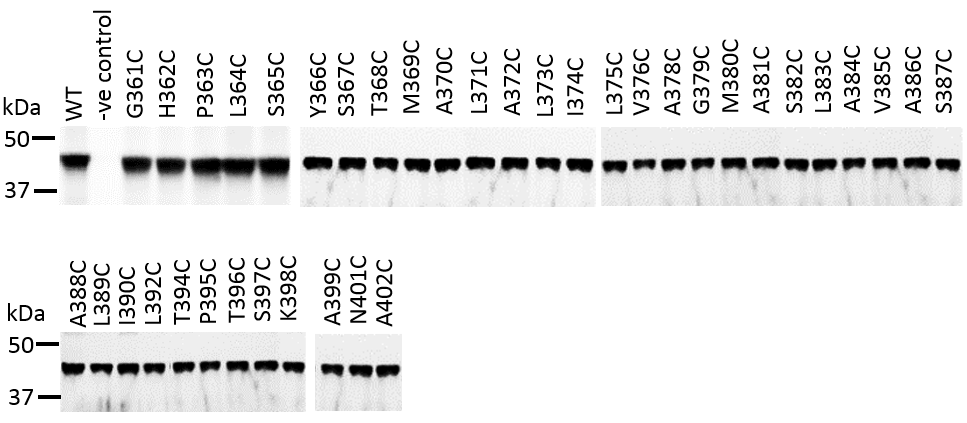


**Figure S1.** Western blot analysis of QacA mutants examined in this study. Protein expression was analysed using membrane vesicles isolated from *E. coli* DH5α expressing wild-type (WT) QacA and mutant derivatives. Equal amounts (100 μg) of total membrane proteins were loaded onto 10% polyacrylamide gels and QacA proteins immunologically detected using a rabbit anti-6xHis antibody. The negative (–ve) control is pBluescript II SK vector which lacks the *qacA* gene. Positions of molecular weight markers are indicated and QacA bands are between 37 and 50 kDa.

## Figure S2


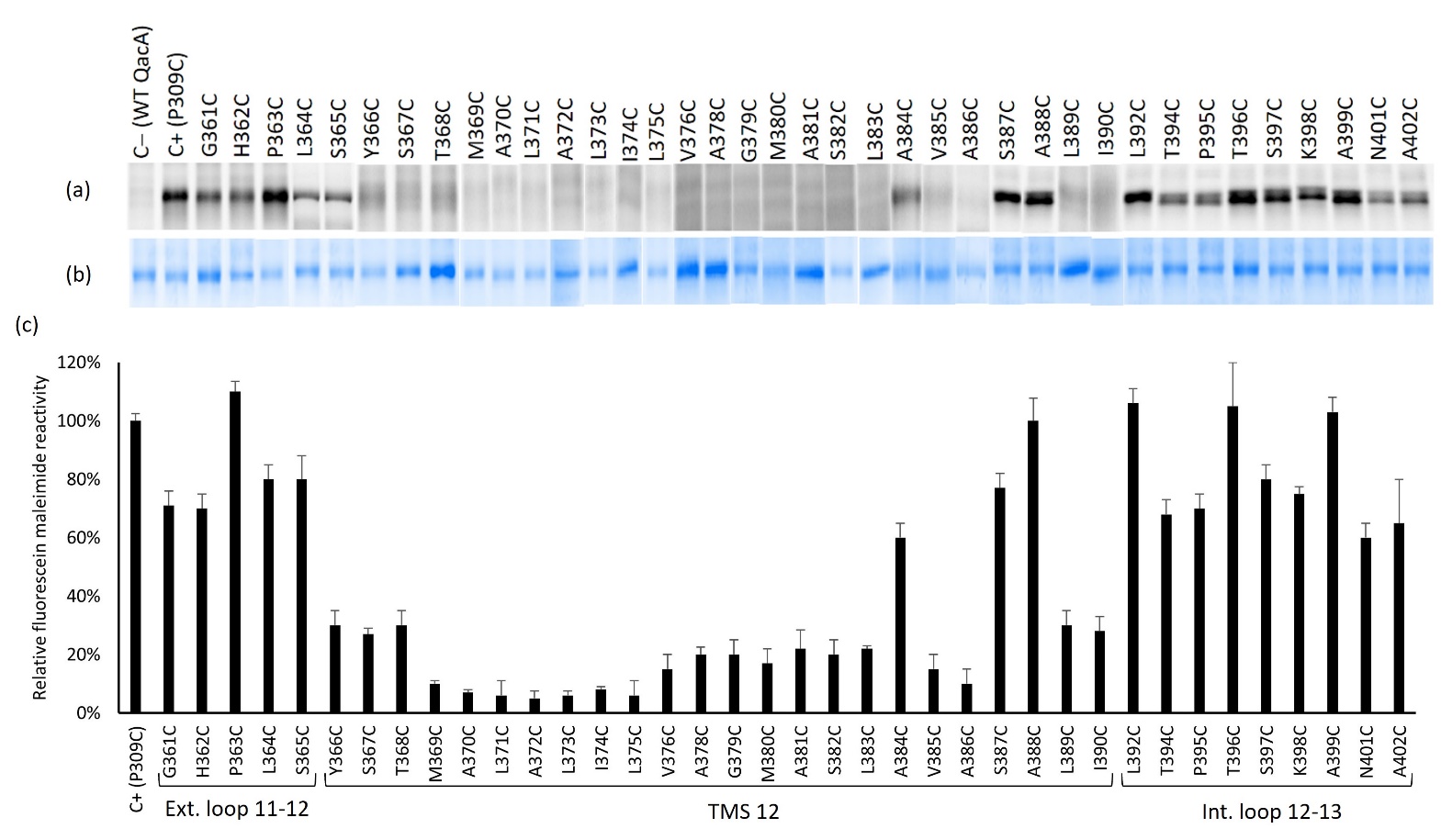


**Figure S2.** Solvent accessibility profile of residues. Membrane vesicles containing single cysteine-substituted QacA mutant proteins were treated with FM and purified by nickel-chelation. Purified QacA proteins were separated on 10 % SDS-PAGE gels. (A) Fluorescence images of labelled QacA proteins visualised with a Gel Doc EZ imager (Bio-Rad). (B) Coomassie Blue staining of SDS-PAGE gels to demonstrate protein amount on gels. (C) The level of fluorescence to the amount of protein for each mutant was quantified densitometrically using ImageLab software (Bio-Rad) and the FM reactivity levels relative to the highly reactive P309C QacA mutant ^1^ are shown. Error bars represent SEM of three biological replicates. Boundaries of TMS 12 are labelled underneath (Ext: Extracellular; Int: Intracellular).

## Figure S3


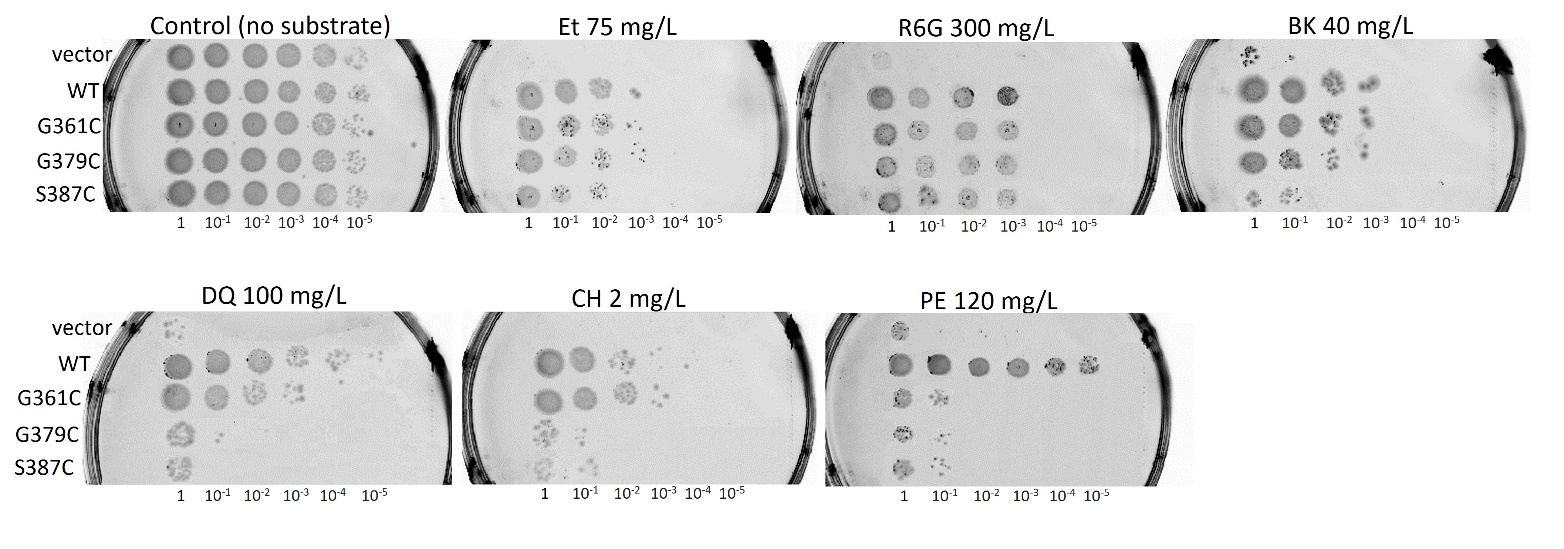


**Figure S3.** Comparison of antimicrobial resistance of *E. coli* cells harbouring empty vector, wild-type QacA (WT) and selected QacA mutants. Plate dilution assays were performed with bacteria harbouring WT and QacA mutants (which conferred ≤50% of wildtype levels of resistance to at least one of the tested bivalent substrates as shown in Table 1) on Mueller-Hinton plates with or without the addition of substrates (concentration given above the plate). Dilution series of overnight cultures with an OD_600_ of 10^0^, 10^-1^, 10^-2^, 10^-3^, 10^-4^ and 10^-5^ were spotted on a Mueller-Hinton agar plate with or without (control plate) the tested antimicrobials. The experiments were conducted at least three times and the results shown are representative.

##
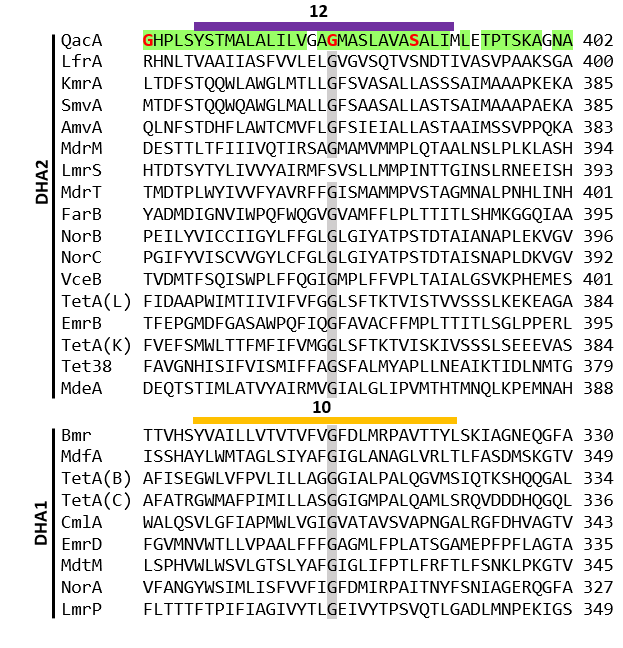
Figure S4

Figure S4. Amino acid sequence alignment of QacA in the region targeted in this study with related MFS DHA1 and DHA2 efflux proteins. Amino acid sequences of selected bacterial DHA1 and DHA2 drug transporters were aligned with QacA using Clustal Omega. Sequences were obtained from the UniProtKB/Swiss-Prot database with the accession numbers as follows: S. aureus QacA (P0A0J8); Mycobacterium smegmatis LfrA (A0R5K5); *K. pneumoniae* KmrA (W9BGM8); Salmonella typhimurium SmvA (D0ZXQ3); *A. baumannii* AmvA (C4PAW9); Listeria monocytogenes MdrM (I0J098); S. aureus LmrS (A0A4T9Z2L7); Listeria monocytogenes MdrT (I0J094); Neisseria gonorrhoeae FarB (Q9RQ29); S. aureus NorB (Q7A5M0); S. aureus NorC (A0A0E1ACG1) Vibrio cholerae VceB (O51919); Bacillus subtilis TetA(L) (P23054); Escherichia coli EmrB (P0AEJ0); S. aureus TetA(K) (P02983); S. aureus Tet38 (Q5PU79); S. aureus MdeA (A0A660A2M0); Bacillus subtilis Bmr (P33449); E. coli MdfA (P0AEY8); Escherichia coli TetA(B) (P02980); E. coli TetA(C) (P02981); *Pseudomonas aeruginosa* CmlA (P32482); E. coli EmrD (P31442); E. coli MdtM (P39386); S. aureus NorA (P0A0J4); Lactococcus lactis LmrP (Q48658). Only a fraction of the alignment encompassing the region targeted in this study is shown. Sequence names are shown on left with their grouping into MFS DHA1 or DHA2 families. Numbers on the right refer to the position of the rightmost residue on each line. Residues targeted for mutagenesis are highlighted in green shading and G361, G379 and S387 identified as functionally important residues in QacA are in red. Glycine residues conserved in positions corresponding to G379 residue in QacA are highlighted in grey. TMS 12 in DHA2 14-TMS members including QacA corresponds to TMS 10 of the DHA1 12-TMS transporters, according to the evolution model of 14-TMS MFS transporter (6+2+6). The location of the TMS 12 in QacA (as determined in Figure 1) and the approximate location of TMS 10 in DHA1 transporters are indicated by horizontal purple and orange bars above the alignment, respectively.

## Table S1

Table S1. Primers used in this study

| Primer*^a^* | Nucleotide sequence (5'→3')*^b^*^,^*^c^* | Restriction e enzyme*^d^* | |
| --- | --- | --- | --- |
| QacA mutagenic primers | | |  |
| G361C_F | GTTTATTATGTATTTCTTT**T**GTCATCC*C*TTA*AGT*TATTCTACAATGGCTT | | *Af*lII |
|  |  | |  |
| H362C_F | GTATTTCTTTGGT**TG**TCC*C*TTA*AG*TTATTCTACAATGGCTTTAG | | *Afl*II |
| P363C_F | GTATTTCTTTGG*C*CAT**TGT**TTATCATATTCTACAATGGC | | *Msc*I |
| L364C_F | GTATTTCTTTGG*C*CATCCAT**GT**TCATATTCTACAATGGC | | *Msc*I |
| S365C_F | GGTCATCCATTAT**GT**TATTCTACAATGGC*GC*TAGCATTAATTTTAG | | *Nhe*I |
| Y366C_F | CTTTGGTCATCC*C*TTA*AGT*T**G**TTCTACAATGGCTTTAGC | | *Afl*II |
| S367C_F | CCATTATCATATT**G**TACAATGGCTTTAGC | | *BsrG*I |
| T368C_F | CCATTATCATATTCT**TGT**ATGGC*GC*TAGCATTAATTTTAGTTG | | *Nhe*I |
| M369C_F | CCATTATCATATTCTACA**TGT**GC*GC*TAGCATTAATTTTAGTTGGAGC | | *Nhe*I |
| A370C_F | CTTTGGTCATCC*C*TTA*AGT*TATTCTACAATG**TGC**TTAGCATTAATTTTAGTTGG | | *Afl*II |
| L371C_F | CATATTCTACAATGGC*A*T**GC**GCATTAATTTTAGTTGGAGC | | *Sph*I |
| A372C_F | CCATTATCATATTCTAC*C*ATGGCTTTA**TGC**TTAATTTTAGTTGGAGC | | *Nco*I |
| L373C_F | CAATGGCTTTAGCAT**GC**ATTTTAGTTGGAGCTGG | | *Nsi*I |
| I374C_F | GGCTTTAGCATTA**TG**TTTAGTTGGAGCTGG*G*ATGGCTTCACTAGC | | *BseY*I |
| L375C_F | GGCTTTAGCATTAATTT**GC**GTTGGAGCTGG*G*ATGGCTTCACTAGC | | *BseY*I |
| Primer*^a^* | Nucleotide sequence (5'→3')*^b^*^,^*^c^* | Restriction e enzyme*^d^* | |
| QacA mutagenic primers | | |  |
| V376C_F | GCATTAATTTTA**TG**TGGAGCTGG*G*ATGGCTTCACTAGC | | *BseY*I |
| A378C_F | GCATTAATTTTAGTTGGA**TGC**GG*A*ATGGCTTCACTAGC | | *Xmn*I |
| G379C_F | GCATTAATTTTAGTTGGAGCT**T**GTATGGCTTC*G*CTAGCAGTTGC | | *Nhe*I |
| M380C_F | GTTGGAGCTGGT**TGC**GCTTC*G*CTAGCAGTTGC | | *Nhe*I |
| A381C_F | GGAGCTGGTATG**TG**TTC*G*CTAGCAGTTGCATCTGC | | *Nhe*I |
| S382C_F | GTTGGAGCTGG*G*ATGGCTT**GC**CTAGCAGTTGCATCTGC | | *BseY*I |
| L383C_F | GTTGGAGCTGG*A*ATGGCTTCA**TGC**GCAGTTGCATCTGC | | *Xmn*I |
| A384C_F | GTTGGAGCTGG*G*ATGGCTTCACTA**TGC**GTTGCATCTGC | | *BseY*I |
| V385C_F | GCTGGTATGGCTTC*G*CTAGCA**TGC**GCATCTGCTCTAATAATG | | *Nhe*I |
| A386C_F | GGTATGGCTTC*G*CTAGCAGTT**TGC**TCTGCTCTAATAATG | | *Nhe*I |
| S387C_F | GGCTTCACTAGCAGTTGCAT**GC**GCTCTAATAATGTTAGAAACACC | | *Sph*I |
| A388C_F | GCTTCACTAGCAGTTGCATCT**TG**TCT*T*ATAATGTTAGAAACACC | | *Psi*I |
| L389C_F | GGCTTCACTAGCAGTTGCATCTGC*A***TGC**ATAATGTTAGAAACACC | | *Nsi*I |
| I390C_F | GCAGTTGCATCTGCTCTA**TGC**ATGTTAGAAACACC | | *Nsi*I |
| L392C_F | GCAGTTGCATCTGCTCT*T*ATAATGT**GC**GAAACACCTACATCAAAAGC | | *Psi*I |
| T394C_F | GCTCTAATAATGTTAGAA**TGC**CCTACATCAAAAGC | | *Bsm*I |

| Primer*^a^* | Nucleotide sequence (5'→3')*^b^*^,^*^c^* | Restriction e enzyme*^d^* | |
| --- | --- | --- | --- |
| QacA mutagenic primers | | |  |
| P395C_F | GCTCTAATAATGTTAGAAACA**TG**TACATCAAAAGCAGG | | *Pci*I |
| T396C_F | GCATCTGCTCT*T*ATAATGTTAGAAACACCT**TGC**TCAAAAGCAGG | | *Psi*I |
| S397C_F | GCTCTAATAATGTTAGAAACACCTACAT**GT**AAAGCAGGTAATGC | | *Pci*I |
| K398C_F | GAAACACCTACATCA**TGT**GCAGGTAATGCAGC | | *Bsg*I |
| A399C_F | CCTACATCAAAA**TGC**GG*G*AATGCAGCTGC | | *Bsm*I |
| N401C_F | CCTACATCAAAAGCAGGT**T*GT***GCAGCTGCTGTTG | | *Bsg*I |
| A402C_F | CAAAAGCAGGTAAT**TGC**GCTGC*A*GTTGAAGAGTC | | *Pst*I |
| A157G_F | GCTTCATCGAT*C*GGTG**G**TGTTTTTGGACCAATTATCG | | *Pvu*I |
| A378V_F | GCATTAATTTTAGTTGGAG**T**TGG*A*ATGGCTTCACTAGC | | *Xmn*I |
| Sequencing primers | | |  |
| M13_F | TGTAAAACGACGGCCAGT | |  |
| M13_R | CAGGAAACAGCTATGACC | |  |
| pBAD_F | ATGCCATAGCATTTTTATCC | |  |
| pBAD_R | GATTTAATCTGTATCAGG | |  |

***^a^*** Each primer is presented by the one-letter amino acid code and its corresponding position number in the QacA amino acid sequence followed by the second letter representing the new generated amino acid.

***^b^*** Primers are presented in a 5’ to 3’ direction. Two primers were used to create each mutation. Only forward (_F) mutagenic primers are listed. The reverse (_R) primers are the reverse and complement of the forward primer sequence.

***^c^*** The nucleotide change(s) leading to the new amino acid replacement are in bold. The nucleotide change(s) that created a silent restriction site mutation are indicated in italics and the restriction sites are underlined.

***^d^*** Enzymes used for restriction digestions to screen for desired mutations prior to sequence verification.

**References:**

**1** Xu Z, O'Rourke BA, Skurray RA et al. Role of transmembrane segment 10 in efflux mediated by the staphylococcal multidrug transport protein QacA. *J Biol Chem* 2006; **281**: 792-9.
